# Global classification of river morphology based on inland water dynamics characterization and digital elevation data

**DOI:** 10.1038/s41598-025-99174-7

**Published:** 2025-04-24

**Authors:** Yilin Li, Yueze Zhang, Naixi Zheng, Lei Li, Hancheng Ji, Zhidong Bao, Zhiqiang Feng

**Affiliations:** 1 State Key Laboratory of Petroleum Resources and Engineering, China University of Petroleum (Beijing), Beijing, 102249 China; 2 College of Geosciences, China University of Petroleum (Beijing), Beijing, 102249 China; 3 Institute of Unconventional Oil and Gas, Northeast Petroleum University, Daqing, Heilongjiang 163318 China; 4 Beijing Key Laboratory of Positive Design and Intelligent Processing Technology for High Precision Machine Tools, Beijing University of Technology, Beijing, 100022 China; 5 Petroleum Exploration and Production Research Institute, Sinopec, Beijing, 102206 China; 6 Deep-Time Digital Earth, Zhejiang Lab, Hangzhou, Zhejiang 311100 China

**Keywords:** River morphology classification, Deep learning, Global river systems, Spatial distribution, ResNet-50, Hydrology, Solid Earth sciences, Petrology

## Abstract

Classifying river morphology is crucial for fluvial geomorphology and hydrology. River morphology reflects hydrodynamic and sedimentary processes, providing critical insights into the diversity of global river systems. This study establishes a global framework for river morphology classification based on remote sensing and topographic data. Using the Global Inland Water Dynamics Characterization dataset and the global digital elevation model ASTER GDEM V3, a river spatial image decomposition process was developed, dividing global river data into tens of thousands of image blocks containing dynamic imagery and elevation information. A ResNet-50 deep neural network was employed to construct an image-elevation fusion classification model, classifying global rivers into five major types: meandering rivers, braided rivers, straight rivers, anastomosing rivers, and anabranching rivers. These types were further divided into 17 subtypes to capture finer morphological variations. The spatial distribution patterns and morphological features of these river types were analyzed, providing a comprehensive understanding of the global distribution of river planforms. This framework advances the knowledge of river systems at a global scale and lays the foundation for future studies in fluvial geomorphology and hydrology.

## Introduction

River classification forms a critical foundation for studying the geomorphic characteristics, formative mechanisms, and evolutionary patterns of rivers. Through the classification of rivers, it is possible to reveal differences in the morphological structure, sedimentary characteristics, and hydrological processes of various river types, thereby providing theoretical support for understanding the diversity and complexity of fluvial geomorphic systems^1^. Existing research indicates that classification methods based on river planform morphology play an important role. Examples include the categorization of meandering rivers, straight rivers, braided rivers, anabranching rivers, and anastomosing rivers. However, most of these studies focus on local or single-scale classification methods, lacking systematic exploration of the global distribution patterns of river morphologies. At the reach scale, there has been no systematic global classification study that integrates river morphology with topographic information.

The study of river classification originated from descriptive research on river geomorphic characteristics, aiming to analyze the processes through which rivers shape landforms and explore the patterns of surface evolution. Early river classification methods were primarily based on qualitative descriptions of river morphology. Leopold and Wolman (1957) classified rivers into three types—straight, meandering, and braided—based on their planform morphology^2^. Rust expanded this classification by introducing the concepts of bifurcation ratio and sinuosity, categorizing rivers into four types: straight, meandering, braided, and anastomosing rivers^3^. Schumm et al. further linked river types to the longitudinal evolution of river channels through their channel evolution models, proposing five longitudinal distribution types to explain the morphological evolution of rivers from their headwaters to downstream reaches^4^.

With the advancement of research, classification methods have gradually transitioned from qualitative descriptions to approaches characterized by quantification, multi-scale analysis, and integration. In 1994, American hydrologist Rosgen proposed a classification framework that integrates river morphology, hydrology, and sedimentary characteristics. This framework categorizes rivers into eight primary types and 94 secondary types based on channel geometric parameters such as sinuosity, longitudinal slope, and bed material size^5^. Frissell et al. introduced a hierarchical scale model that classifies rivers into nested spatial levels, ranging from watersheds to geomorphic units^6^. This concept was further developed in the European Union’s REFORM (REstoring rivers FOR effective catchment Management) project^7^, which conducted river classification studies based on multi-scale spatial units, such as landscape units, river segments, and geomorphic units.

Although localized studies have provided detailed frameworks and case analyses for river classification, the majority of research remains focused on specific regions or single scales. Examples include the hydromorphological classification of Slovenian rivers^8^, the morphological study of the Rapti River in Nepal^9^, research on rivers in the Peruvian Amazon^10,11^, the planform changes in the lower Mahaweli River^12^, river morphology classification in Ethiopia^13^, and studies on the Brahmaputra River in Bangladesh and Assam, India^14–16^. These studies employ regional classification methods, offering a wealth of scientific evidence for river morphology classification and revealing the complex relationships between river morphology and geomorphic, ecological, or social factors from a regional perspective. However, these efforts typically focus on localized or single-scale studies, limiting the applicability of their classification frameworks. Earlier studies on river morphology classification primarily relied on satellite imagery and did not consider topographic information, except in quantifying valley confinement. To date, systematic studies investigating the hierarchical distribution of river morphology at the reach scale on a global scale, combined with topographic information, remain lacking.

In recent years, the rapid development of remote sensing technology and geographic information science has provided significant support for global river classification studies. According to Belletti, remote sensing data have become the primary source for monitoring river morphology, accounting for as much as 73% of the data used^17^. For example, the long-term multispectral data provided by the Landsat program allow researchers to capture the dynamic changes and spatial distribution patterns of rivers^18,19^. Digital Elevation Model (DEM) data, such as the ASTER GDEM and the SRTM DEM, enable the extraction of key geomorphic parameters, including the elevation distribution of riverbanks and slope, which are closely related to river planform morphology^20,21^. Additionally, research on global terrain classification has further supported efforts in river classification. For instance, Iwahashi et al. used 30-m DEM data to classify the terrain of Japan’s alluvial plains and mountains^22^ and later extended this terrain classification to a global scale^23^.

The emergence of automated classification methods has significantly improved the efficiency and accuracy of river classification, providing technical support for river morphology studies on a global scale^24^. Current automated classification methods can be broadly divided into two categories: non-deep learning-based methods and deep learning-based methods^25^. The former includes techniques such as support vector machines^26^, classification trees^27^, and cluster analysis, which have been applied to regional river morphology classification studies^25^. However, these methods face limitations in efficiency and accuracy when handling large-scale datasets. Deep learning-based methods, such as convolutional neural networks, demonstrate stronger feature extraction capabilities and superior classification performance. For example, Carbonneau et al. utilized deep learning methods to classify river scenes^28^, while Liu et al. applied a scale-aware deep reinforcement learning approach to classify high-resolution remote sensing images^29^. Mukonza et al. used multilayer perceptrons to classify the trophic states of freshwater reservoirs in Taiwan^30^. Although these studies highlight the significant potential of deep learning in river classification, current research remains largely focused on localized areas. To date, there is a lack of classification frameworks that integrate river morphology and topographic information at a global scale.

To address the aforementioned research gap, this study proposes a global river morphology classification method based on deep learning. By combining the Global Surface Water Dynamics dataset with the ASTER GDEM V3 elevation data, river imagery is extracted, and an image-elevation fusion classification model is constructed through a decomposition process of river imagery. Using the ResNet-50 deep neural network and clustering methods, the study achieves a hierarchical classification of river morphologies. By integrating river characteristics with terrain information, this framework analyzes the spatial distribution of river morphologies at the reach scale. The significance of this study lies in its effort to fill the gap in global reach-scale river morphology classification, with a particular focus on the spatial distribution of river types. By establishing a hierarchical classification framework, this research contributes to a deeper understanding of the diversity and heterogeneity of river systems worldwide.

## Methodology

**Fig. 1:**
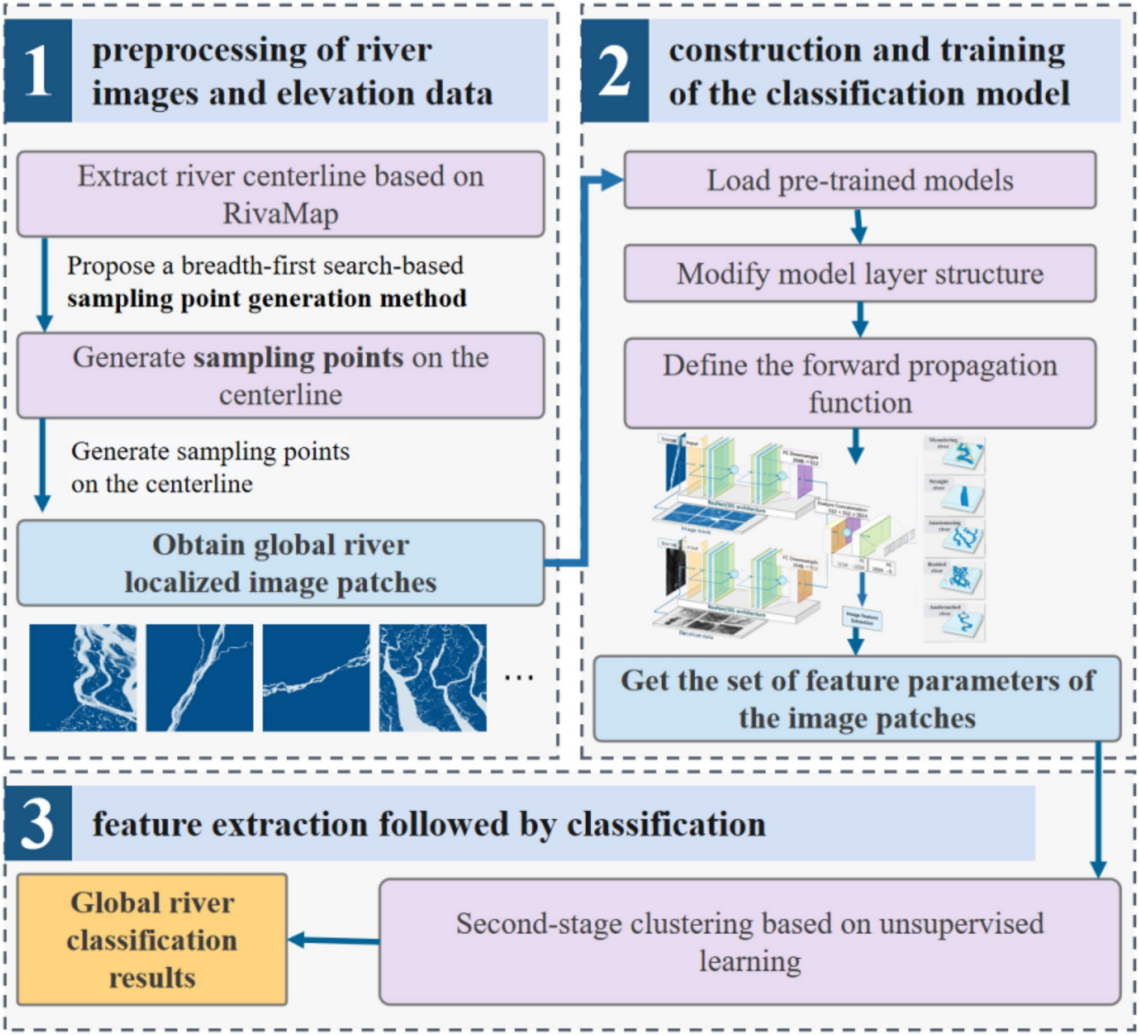
Workflow of the global river morphology classification methodology.

The classification of river morphology features on a global scale is carried out in three steps: preprocessing of river spatial images and elevation data, construction and training of the classification model, and feature extraction followed by classification. In the first step, river centerlines are extracted using RivaMap^31^, an automated river analysis and mapping engine, and sampling points are generated along the centerlines. These points are used to segment and crop river images and corresponding elevation data, creating smaller, well-structured image blocks for the sample set. In the second step, a classification model based on image-elevation fusion is developed and trained using labeled river-image blocks. The fusion model integrates river morphology features from both spatial images and elevation data to improve classification accuracy. In the third step, the trained model is applied to all river images and elevation data for large-scale classification. A second-stage unsupervised clustering refines the results, producing the final global river classification, as illustrated in Fig. 1.

### Dataset acquisition

**Fig. 2:**
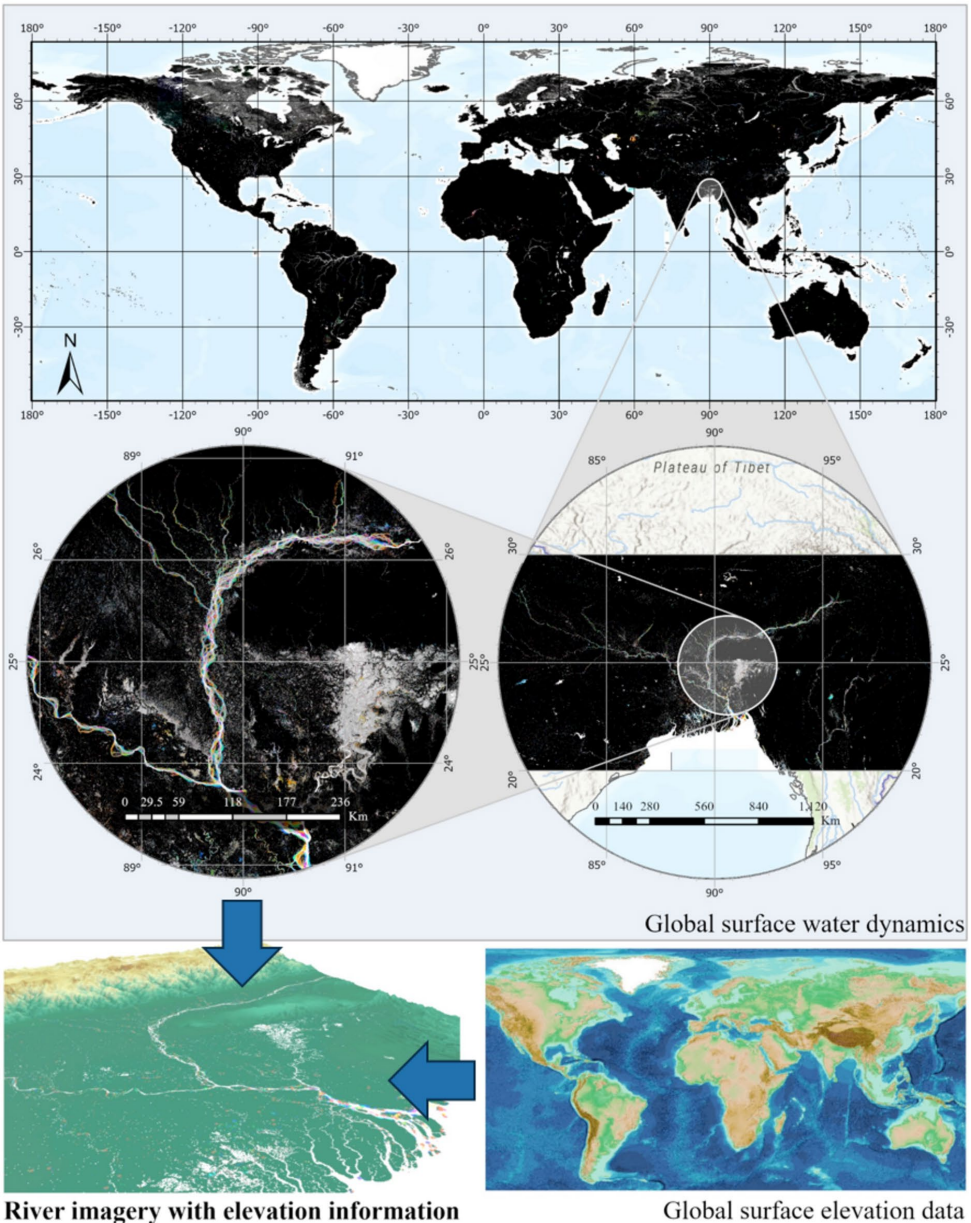
A localized zoomed-in view of the Global Inland Water Dynamics Characterization dataset, with the Bay of Bengal as an example. (The map was generated using ArcGIS Pro version 3.4.0, a desktop GIS software. More information about the software is available at https://www.esri.com/en-us/arcgis/products/arcgis-pro/overview).

This study utilizes the Global Inland Water Dynamics Characterization dataset^32^ and the ASTER GDEM V3 Global Digital Elevation Model^33^ for the training and classification of the river model. The global inland water dynamics characterization dataset is based on Landsat 5, 7, and 8 time-series images from 1999 to 2021, which were used to extract information on global inland water bodies. The dataset defines the “percentage of water bodies” as a quantitative index representing the frequency of occurrence of water bodies. It constructs an annual time series of the percentage of water bodies for each pixel, calculates the variation range, and determines the mean value. Using these parameters, the dataset classifies pixels into three categories: permanent land, permanent water, and dynamic water bodies. For dynamic water bodies, the time series data are mapped to the RGB color space using specific coding rules: the red, green, and blue channels represent the start, mean, and end of the observation cycle, respectively—or the first, middle, and last extremes during two water body transitions. These coding rules allow the visualization of different water body dynamics through distinct color features. The dataset divides the Earth into 10° × 10° grid areas based on latitude and longitude, extracting water body information zone by zone and performing RGB mapping. As shown in Fig. 2, a localized zoomed-in view of the data, with the Bay of Bengal as an example, illustrates this process.

Meanwhile, the ASTER GDEM V3 elevation data provides critical topographic information for this study. Jointly developed by NASA and the Ministry of Economy, Trade, and Industry (METI) of Japan, this dataset is derived from ASTER stereo imagery and features a spatial resolution of approximately 30 m, covering latitudes from 83°N to 83°S. This broad coverage is particularly important for including high-latitude rivers in the study, ensuring the comprehensiveness of the global-scale analysis. Moreover, the ASTER GDEM V3 dataset, released on August 5, 2019, includes an additional 360,000 optical stereo image pairs compared to the previous version (V2). These improvements reduce elevation voids and address anomalies in water body elevation values, enhancing the reliability of this dataset for hydrological and geomorphological studies. In this study, ASTER GDEM V3 was utilized to extract geomorphological parameters, such as elevation distribution and slope on both sides of the river, thereby supplementing the two-dimensional information provided by the inland water characterization dataset. The specifications of the two datasets used in this study are summarized in Table 1.

**Table 1:** Geospatial datasets used in this study.

Dataset name	Source/Provider	Specifications
Global Inland Water Dynamics Characterization dataset^32^	Global Land Analysis & Discovery (GLAD), University of Maryland	Temporal coverage: 1999–2021; Resolution: Landsat-derived, ~ 30 m spatial resolution
ASTER GDEM V3^33^	NASA and Japan’s Ministry of Economy, Trade, and Industry (METI)	Temporal coverage: 2000–2009; Spatial resolution: 30 m; Vertical Datum: WGS84/EGM96

### Preprocessing of river spatial images and elevation data

**Fig. 3:**
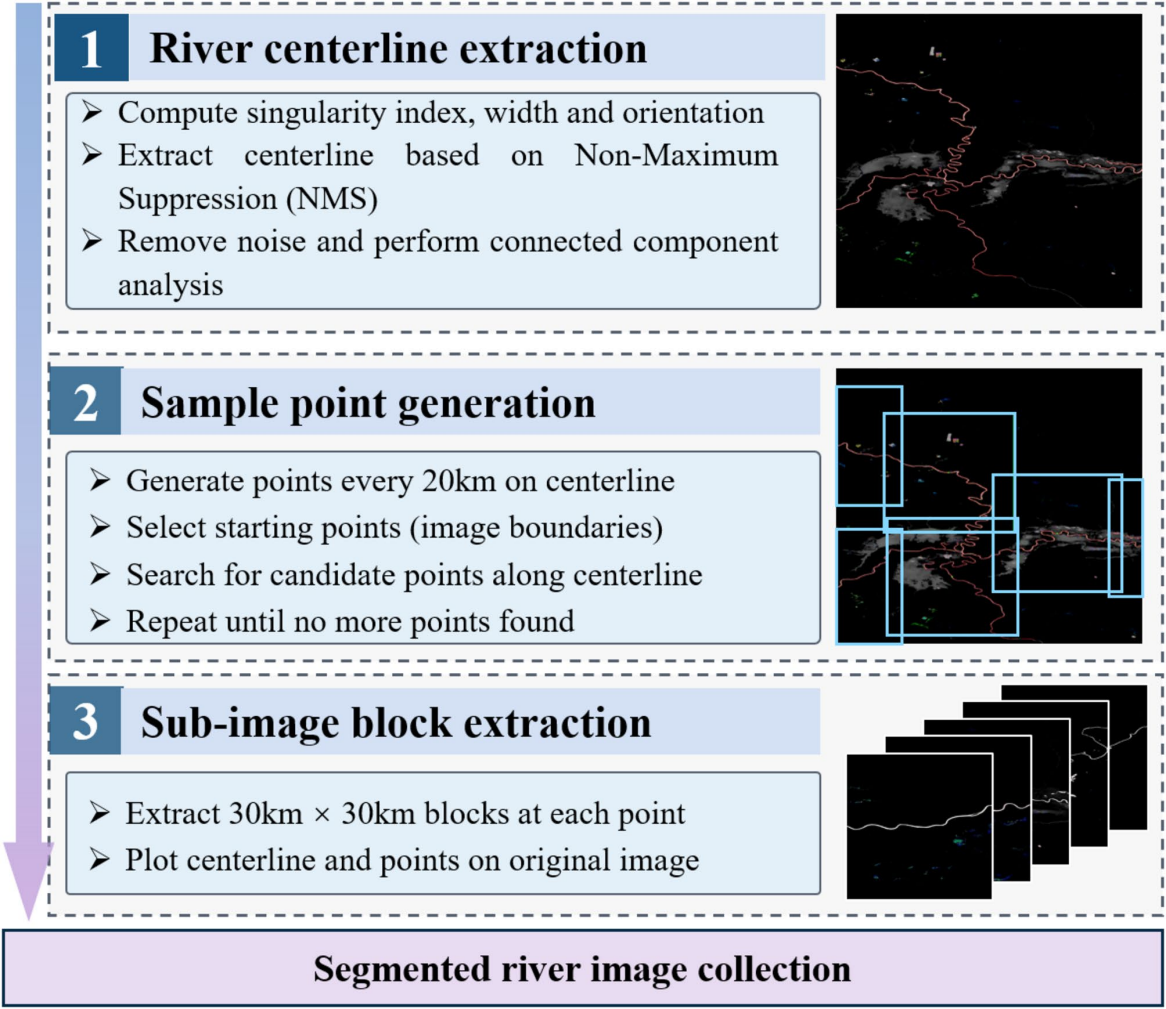
Data source preprocessing: river spatial image decomposition process.

After obtaining the original dataset, this study utilizes RivaMap to develop a river spatial image decomposition process. This process automatically extracts river centerlines and generates evenly distributed sampling points. Each image in the dataset is then cropped according to these sampling points and discretized into a sample set of fixed-size river image patches, as illustrated in Fig. 3.

First, an improved multi-scale singularity index method is used to calculate the singularity index for extracting river centerlines^34^. The singularity index is highly responsive to curvilinear structures. The second-order derivative in the numerator is significant, while the first-order derivative in the denominator shows a weaker response at the edges. Because rivers exhibit distinct curvilinear features, the singularity index effectively extracts river centerlines from remote sensing images. This index can be applied to any image with water-land contrast, such as multispectral remote sensing images or water index maps.

1 $$\left( {{{\uppsi }}f} \right)\left( {xy,{{\upsigma }}} \right) = \frac{{\left| {f_{{\left( {0,{{\uptheta }},{{\upsigma }}} \right)}} \left( {x,y} \right)f_{{\left( {2,{{\uptheta }},{{\upsigma }}} \right)}} \left( {x,y} \right)} \right|}}{{1 + \left| {f_{{\left( {1,{{\uptheta }},a{{\upsigma }}} \right)}} \left( {x,y} \right)} \right|^{2} }}$$

In Eq. (1), $\:{f}_{\left(0,\theta\:,\sigma\:\right)}\left(x,y\right)$, $\:{f}_{\left(1,\theta\:,a\sigma\:\right)}\left(x,y\right)$, and$\:{f}_{\left(2,\theta\:,\sigma\:\right)}\left(x,y\right)$ represent the response values at pixel position$\:\left(x,y\right)$. These values are obtained by applying the zero-order, first-order, and second-order derivatives of the Gaussian function in the $\:{\uptheta\:}$ direction, respectively. Here, $\:{\upsigma\:}$ denotes the scale parameter of the Gaussian function, controlling its smoothing degree. $\:\theta\:\left(x,y\right)$ represents the estimated direction of the derivative at the pixel position $\:\left(x,y\right)$. To estimate the direction $\:\theta\:\left(x,y\right)$, the second-order derivative response at the pixel position $\:\left(x,y\right)$ is calculated. This identifies the direction where the response reaches a local maximum in the neighborhood. This direction is orthogonal to the curvilinear singularity, which is the river centerline in the input image.

This study calculates the singularity index response at multiple scales to capture the features of the river at different hierarchical levels, including the main channel and its tributaries. The information across different scales is integrated to obtain a comprehensive singularity index response by calculating the Euclidean norm of the multi-scale singularity index responses. Next, the Non-Maximum Suppression (NMS) algorithm is applied to each pixel along the dominant direction. By comparing each pixel’s singularity index response values with those of its neighboring pixels and retaining the local maximum response, the NMS algorithm effectively refines and optimizes the extraction of the river centerline. However, the extracted results include centerlines of non-target water bodies such as lakes and swamps. This study employs connected component analysis for post-processing the extracted centerline to eliminate noise from non-target water bodies in the extracted results. To filter out noise and small non-target components, connected regions below a certain size threshold are removed. This filtering step effectively removes non-river centerline components from the extraction results. Subsequently, a method for generating river centerline sampling points based on Breadth-First Search (BFS) is developed, as shown in Algorithm 1.

**Algorithm 1:**
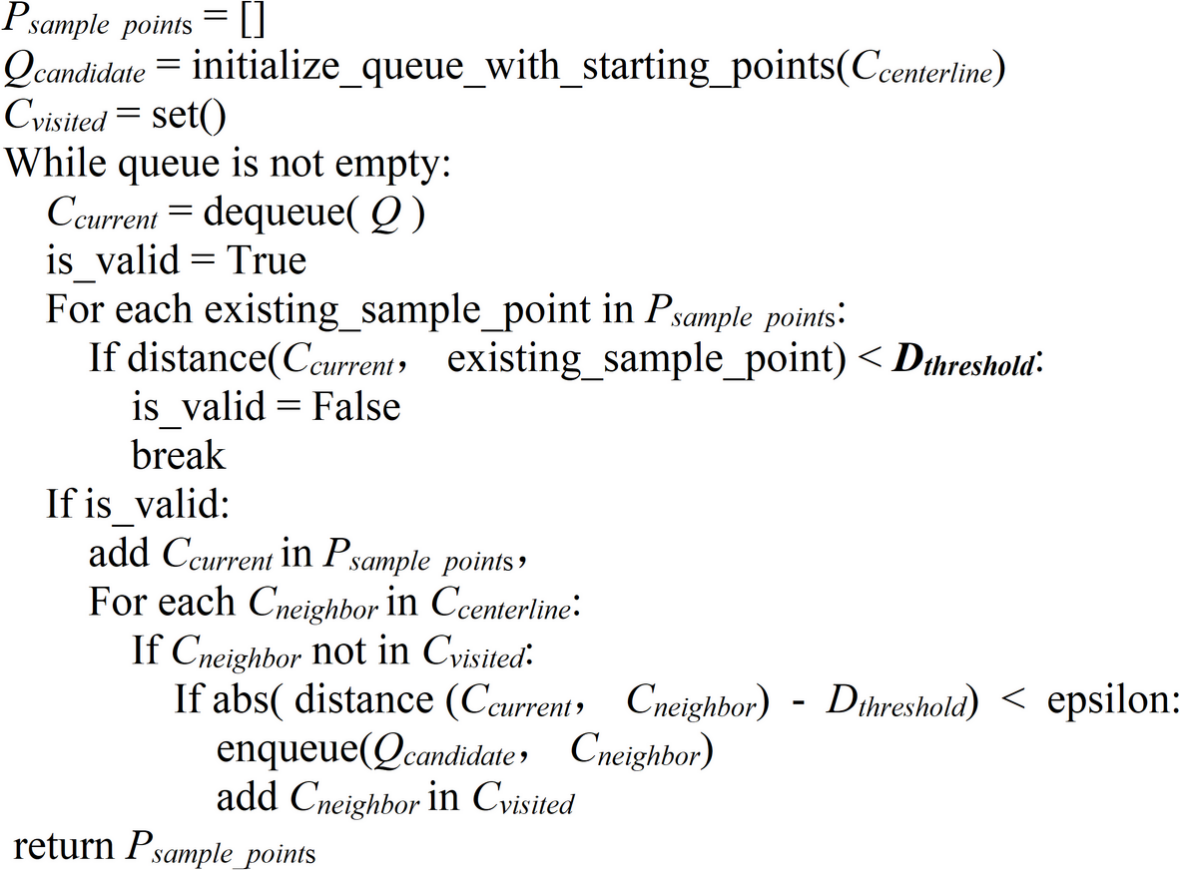
Function generate_sample_points (*C*_*centerline*_, *D*_*threshold*_, *epsilon*)

The algorithm takes the river centerline coordinate set *C*_*centerline*_ and the distance threshold *D*_*threshold*_ as inputs to generate a set of uniformly distributed sampling points *P*_*sample_point*s_. The algorithm initializes the sampling point list, candidate point queue, and visited coordinates set. The search starts from the topmost, bottommost, leftmost, and rightmost boundaries of the river centerlines. Using the BFS strategy, the algorithm incrementally explores candidate points on the river centerline. For each candidate point, the algorithm checks if it meets the distance threshold condition relative to the existing sampling points. An equidistant sampling strategy is adopted to ensure a uniform distribution of sampling points, with one point generated every 20 km along the river’s length. If the condition is met, the point is added to the sampling point list, and the search continues for its neighbors on the centerline within the *D*_*threshold*_ distance. Points that do not meet the condition are added to the set of visited coordinates. The algorithm avoids redundant visits and computations by maintaining the visited coordinates set. The search process continues until the candidate point queue is empty. Finally, the algorithm returns the generated list of sampling points.

A square area measuring about 30 × 30 km was extracted from the original imagery, centered on each sampling point. This square area constitutes a single sub-image block. The 30-km scale was chosen to ensure that each sub-image block typically contains relatively uniform river features, minimizing the mixing of multiple feature types. At this scale, the focus is on capturing curvature changes in channel morphology, spatial distribution patterns of tributary networks, and associated river features. Subsequently, several sub-image blocks were cropped from each river image in the dataset to characterize global inland-water dynamics. Simultaneously, the elevation data for the same region were cropped, and the two datasets were paired to form the initial sample set for river classification.

### Image-elevation fusion classification model construction and training

To achieve the fusion classification of river image data and elevation data, this study develops an image-elevation fusion classification model based on a deep learning framework, which is subsequently trained and optimized. The model is built upon a convolutional neural network (CNN) and adopts a two-branch structure to separately process image features and elevation features. The image branch extracts spatial and texture information from river images, while the elevation branch processes elevation data to capture topographic features and height variations. A feature fusion module is then employed to integrate the multi-source information, enabling joint characterization and providing input support for the classification task.

The first part of the model is a two-branch feature extraction module. The imaging branch utilizes ResNet-50^35^ as the backbone network to extract high-dimensional features from the image data. ResNet-50 was chosen for its deep feature learning capability and innovative architecture, which addresses the vanishing gradient and degradation problems common in deep neural networks. This model, comprising 50 layers, utilizes residual units with skip connections to effectively train very deep networks by preserving the original input information while learning new features. The image input consists of three channels (RGB), and after convolution and pooling operations, the output is a one-dimensional feature vector that captures the spatial distribution and texture information of the image.

**Fig. 4:**
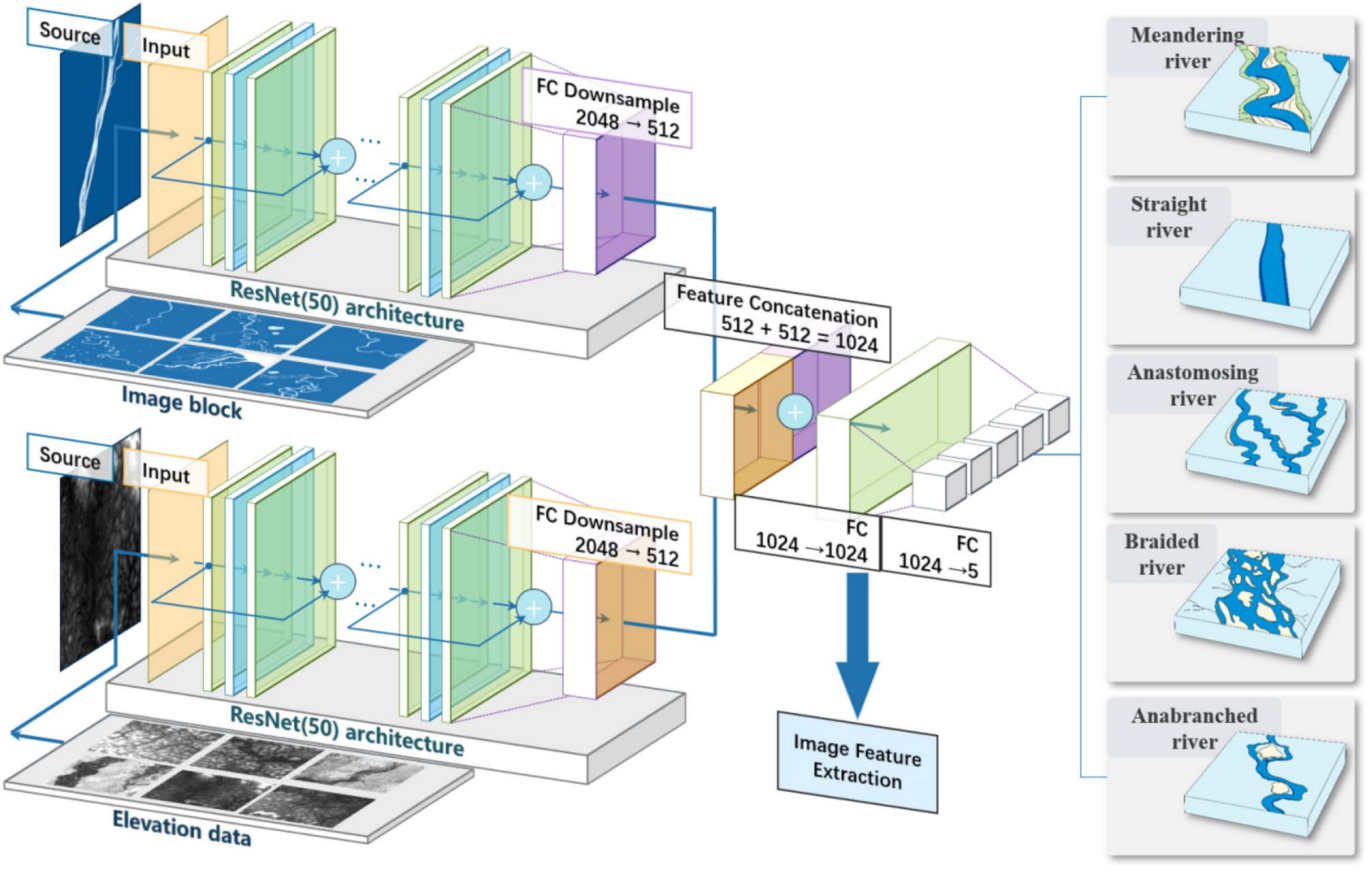
Architecture of the modified ResNet-50 neural network used for river image-elevation fusion classification.

The second part of the model is the feature fusion and classification module. High-dimensional features output from the image branch and elevation branch are first downscaled to the same dimension using a fully connected layer. These features are then concatenated to form a joint feature representation, effectively integrating image and elevation information. The fused features are passed through a two-layer fully connected classifier to produce the final classification results. In this process, the first fully connected layer enhances the feature representation through nonlinear transformations, while the second fully connected layer generates the probability distributions for the five river types. The Rectified Linear Unit (ReLU) activation function is employed in the classifier to facilitate nonlinear feature learning and improve the model’s representational capacity, as shown in Fig. 4.

The model is designed not only to support the classification task but also to function as a feature extractor. By freezing the trained parameters, the model can extract the joint feature representation after processing the image and elevation data. These extracted features can then be utilized for unsupervised clustering analysis.

### Model training

This study adopted a classification framework based on river planform morphology, categorizing rivers into five types: meandering, straight, anastomosing, braided, and anabranching. This classification scheme is grounded in extensive literature while accounting for the diversity of river morphological characteristics and the practical requirements of global-scale classification. The classification of river planform morphology was first proposed by Leopold and Wolman, who divided rivers into three types: straight, meandering, and braided rivers^2^. Rust later expanded this framework to include four types—straight, meandering, braided, and anastomosing rivers—by introducing branching and sinuosity indices^3^. Nanson and Knighton^36^ further proposed the concept of anabranching rivers, defining them as multi-channel systems formed under varying energy regimes, with anastomosing rivers considered a subset.

**Fig. 5:**
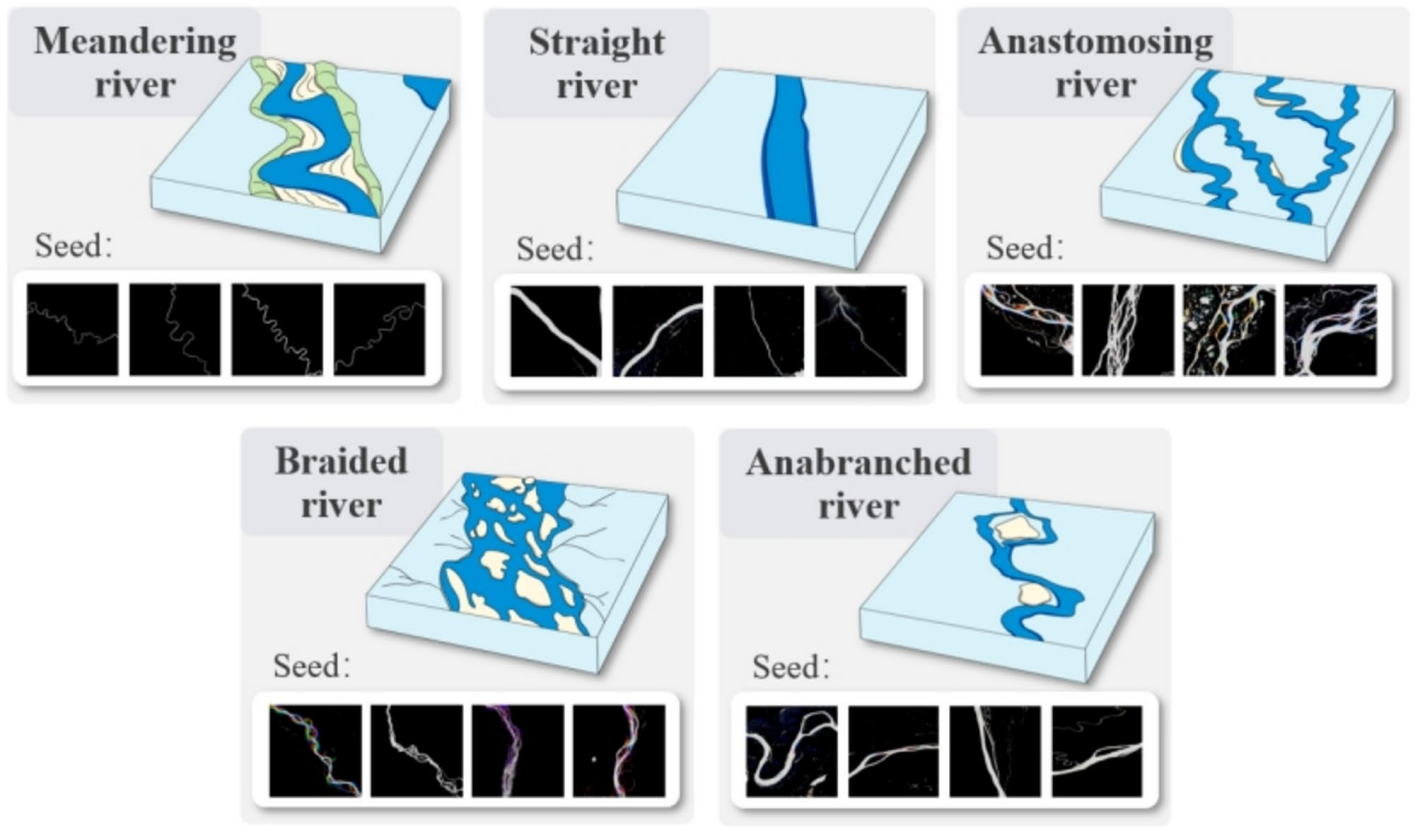
Five primary river types used in this study, classified based on planform morphology.

However, anastomosing rivers exhibit unique geomorphological characteristics and stability. Specifically, anastomosing rivers represent low-energy equilibrium systems with large bifurcation angles (close to obtuse angles), high channel stability, and minimal lateral migration^36–40^. In contrast, anabranching rivers, as a broader category, have smaller bifurcation angles and exhibit greater variability in the stability of channels and islands, reflecting a range of energy conditions from low to high^37,38^. Furthermore, considering that this study utilizes global river dynamic imagery as its primary data source, riverbank stability was another important factor supporting the independent classification of anastomosing rivers. For model training, river image blocks exhibiting the typical characteristics of these five river types were selected from the dataset and used as training labels, as shown in Fig. 5.

The model training is conducted using a supervised learning strategy, with the cross-entropy loss function serving as the objective function. The optimization process employs the Adam optimizer^41^, with an initial learning rate of 0.0001. The training dataset consists of 10,000 manually labeled image and elevation data pairs, which are split into a training set, validation set, and test set in a ratio of 8:1:1. The task is to classify river images into the mentioned five types. During training, the data are fed into the two-branch feature extraction module of the model. The image branch and elevation branch extract features using ResNet-50, after which the feature fusion module generates joint features for classification prediction. The model is trained for a maximum of 200 iterations (training cycles) with a batch size of 16. In each training cycle, the model parameters are updated using the training set, while performance is evaluated on the validation set. To prevent overfitting, the model weights corresponding to the highest validation accuracy are saved. After training is complete, the model, loaded with the optimal weights, is evaluated for final performance, achieving a classification accuracy of 96.52%.

### Hierarchical clustering process

**Fig. 6:**
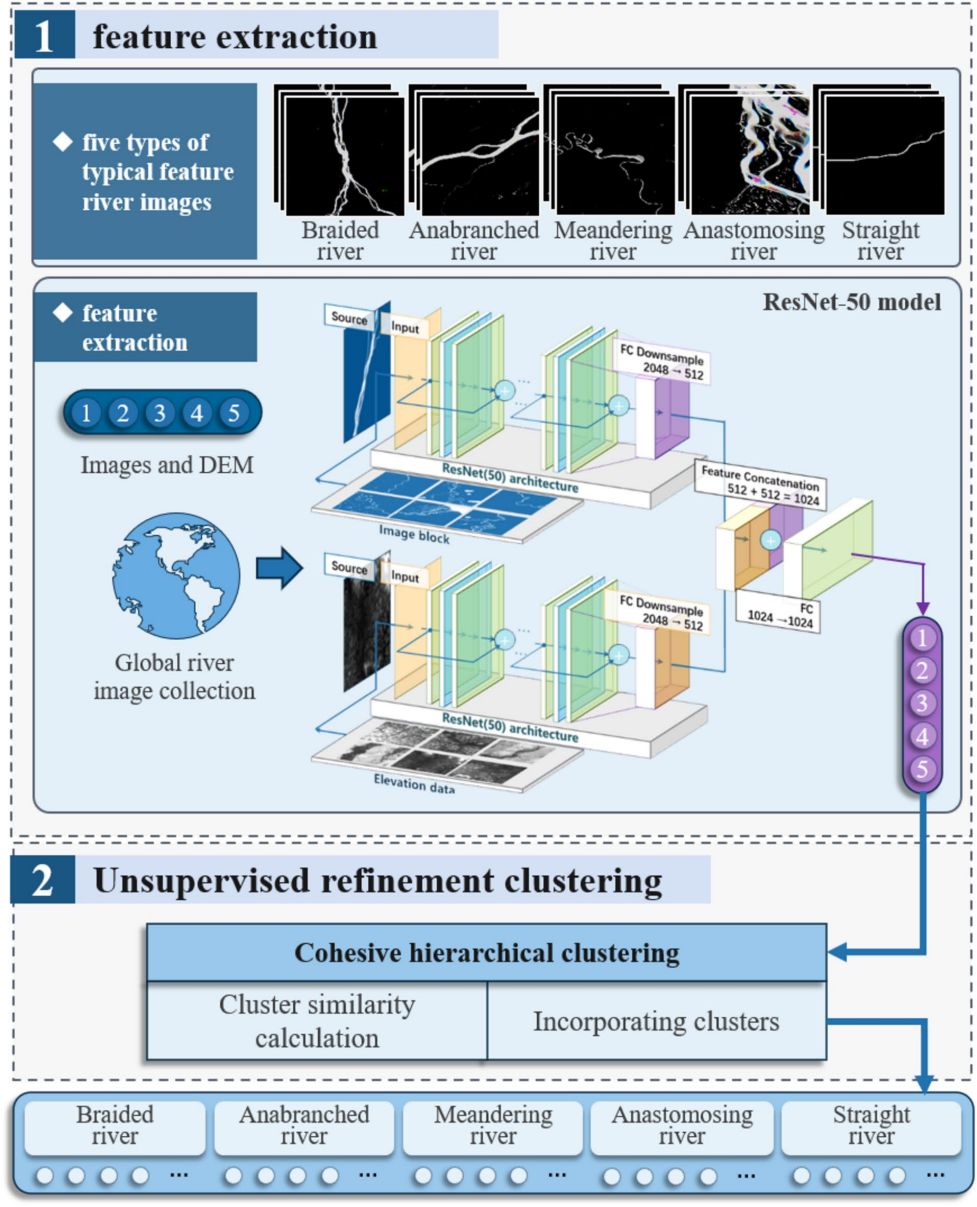
Flowchart of hierarchical clustering for river subtype classification.

Rivers exhibit significant variations in their geomorphic and hydrological characteristics, influenced by factors such as regional topography, sediment supply, and hydrological conditions. To capture these differences, fine-grained classification is essential for revealing the hierarchical structure of river morphologies. After obtaining the initial sample set for river classification, this study employs a cohesive hierarchical clustering algorithm to further refine river features. The algorithm first extracts river image block features using a previously trained network. Based on the initial typical types—meandering, straight, anastomosing, braided, and anabranching—the algorithm further subdivides these categories into subtypes, uncovering detailed hierarchical structures within the river features. The hierarchical clustering process is illustrated in the flowchart in Fig. 6.

The first stage, feature extraction, utilizes a pre-trained model to classify river images into the five primary categories. Each type is treated as an independent dataset, and the pre-trained model is used again to extract image features for each category. Then, in the second stage, an unsupervised hierarchical clustering algorithm is applied to conduct fine-grained clustering within each type’s collection of river images. Hierarchical clustering is a bottom-up algorithm that initially treats each sample’s high-dimensional vector as an independent cluster. The algorithm progressively merges the most similar clusters until a predetermined number of clusters is reached or specific termination conditions are satisfied. The merging sequence is determined by calculating the similarity or distance between clusters. This unsupervised fine-grained clustering stage aims to uncover potential internal structures and variations within each type, identifying possible subtypes or variants. The granularity and outcomes of the refined clustering are controlled by adjusting the distance threshold parameters of the clustering algorithm.

Ultimately, through this two-stage clustering process, the study achieves refined classification results for rivers globally, providing a comprehensive and detailed understanding of river morphologies worldwide.

### Methods of river analysis

Building on the classification results obtained through hierarchical clustering, this study focuses on analyzing the geometric characteristics of river morphology and associated topographic features. To investigate elevation changes on both sides of rivers and combine river centerline data with elevation image data, a method was developed. Virtual transect lines were generated perpendicular to the river’s flow direction at regular intervals along the river centerline. Each transect extended equally on both sides of the river, with a total length of 2 km (1 km on each side), and elevation data were sampled at 50-meter intervals along the transect.

The river centerline was first extracted from river network data. Sampling points were then generated along this centerline at regular intervals of 50 pixels. For each sampling point, the tangent direction of the river centerline was determined using the RivaMap tool, and a transect line perpendicular to this tangent direction was drawn through the sampling point. The geographic coordinates of the sampled points along each transect were calculated using the following formulas:

2 $$\begin{gathered} \Delta {\text{lat}} = \frac{{{\text{distance}}}}{{R_{{{\text{Earth}}}} }} \cdot \sin \left( {{\text{direction}}} \right) \hfill \\ \Delta {\text{lon}} = \frac{{\frac{{{\text{distance}}}}{{R_{{{\text{Earth}}}} }} \cdot \cos \left( {{\text{direction}}} \right)}}{{\cos \left( {{\text{center\_lat}}} \right)}} \hfill \\ \end{gathered}$$

Here, $\Delta {\text{lat}}$ and $\Delta {\text{lon}}$ are the increments of latitude and longitude, respectively; distance is the spacing between points along the transect; *R*_Earth_ is the radius of the Earth; direction is the direction angle of the transect line (in radians); and *center_lat* is the latitude of the river center point. Using these formulas, the geographic coordinates of all points along the virtual transect line were iteratively calculated, encompassing both the left and right banks of the river.

After obtaining the geographic coordinates of the transect points, elevation data corresponding to each point were extracted from GeoTIFF elevation images. The transect was divided into left and right banks based on the center point, and elevation profiles were generated for both banks. Along each transect, the slopes of the left and right banks were calculated by fitting a linear regression to the elevation data points on the respective side of the river centerline. The slope curves for all transects were then summed and averaged to generate the final average slope curves for the left and right banks in the analyzed region. To standardize the elevation profiles for inter-regional comparisons, the elevation curves were normalized by taking the centroid elevation of each transect as the benchmark and calculating relative elevation changes. This process yielded standardized cross-sectional profile curves for both the left and right banks.

This method may produce transect profiles that appear overly simplified or triangular, which differs from the more detailed profiles observed in smaller-scale studies. This discrepancy arises due to the spatial extent of the elevation data and the scaling required for large-scale analysis. This method is well-suited for large-scale investigations, as it effectively reflects the general topographic characteristics of riverbanks.

## Analysis of river-type clustering results

### Distribution of river types and quantities

The global inland water dynamics characterization dataset used in this study consists of raw image blocks, each with a resolution of 20,000 × 20,000 pixels, representing a 10° × 10° region in latitude and longitude. To optimize computational efficiency, the raw data were initially cropped into subregions, each covering a 2° × 2° range in latitude and longitude, resulting in image blocks with a resolution of 4,000 × 4,000 pixels. This process yielded a total of 1,710 image blocks, representing global water coverage, along with corresponding elevation data blocks.

Using a river spatial image decomposition process, rivers were further cropped based on their centerlines, resulting in the extraction of 46,822 local river image blocks, each covering approximately 30 km. Corresponding local elevation data blocks were extracted using the same regions. Subsequently, rivers were classified into five distinct types through a supervised model-based labeling process. This classification was performed as the first step in analyzing river morphology, ensuring that each image block was assigned to one of the five primary river types.

**Fig. 7:**
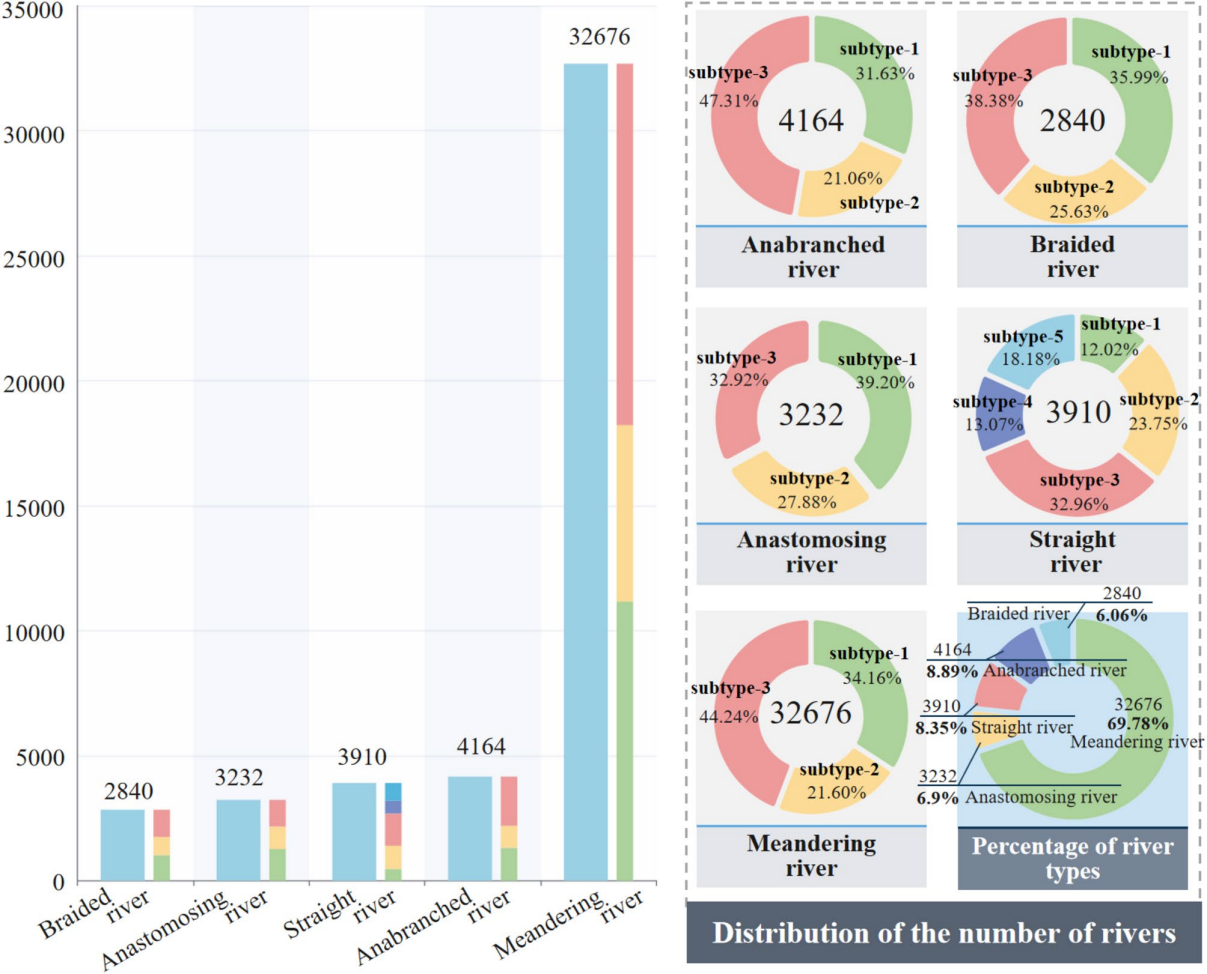
Quantitative and proportional distribution of the five major river types in the dataset.

The classification results indicated that meandering rivers were the most prevalent, accounting for 69.78% of the dataset (32,676 images). Straight rivers accounted for 8.35% (3,910), braided rivers accounted for 6.06% (2,840), anastomosing rivers accounted for 6.90% (3,232), and anabranching rivers accounted for 8.89% (4,164), as shown as Fig. 7.

**Fig. 8:**
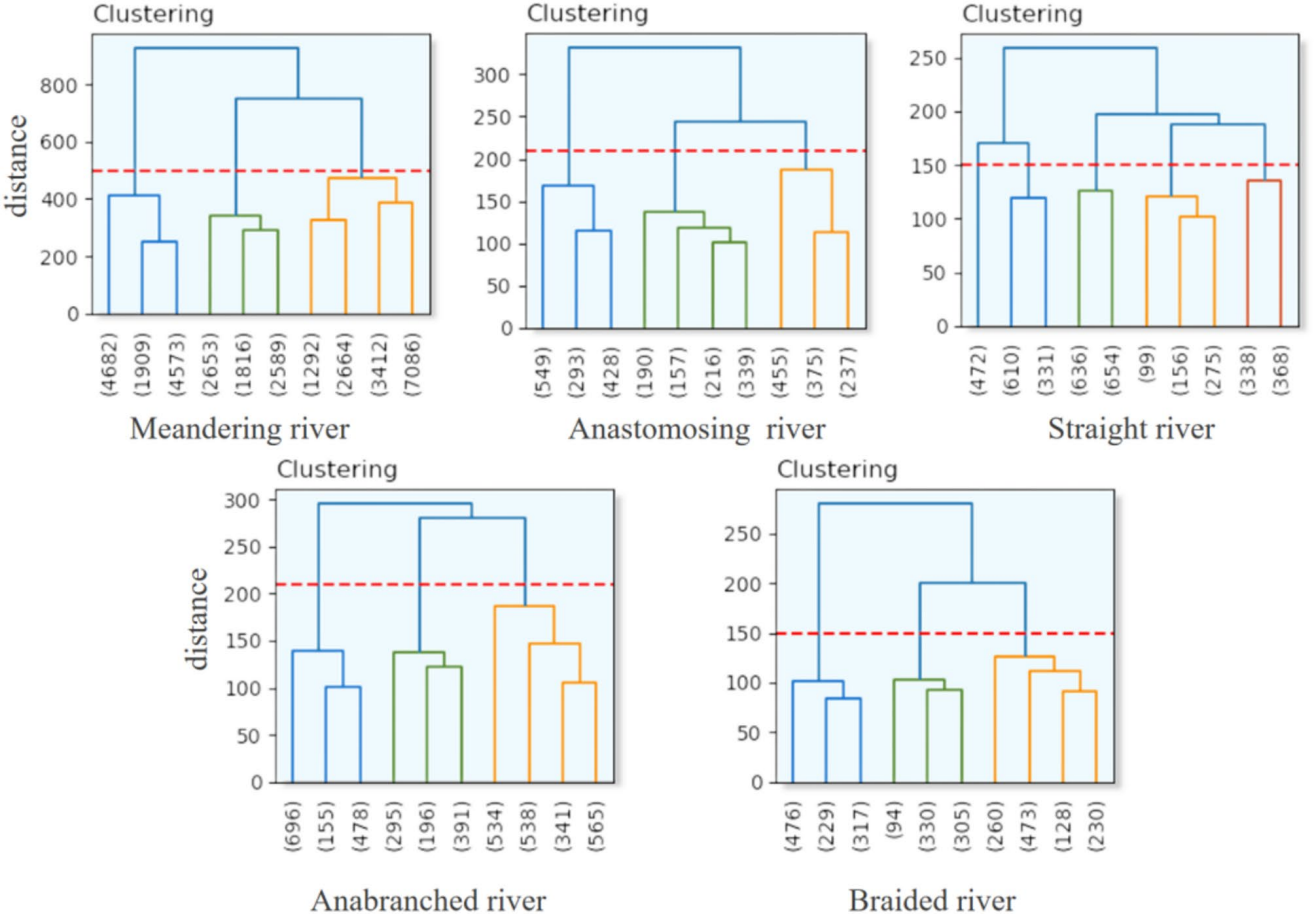
Hierarchical clustering diagram of river subtypes.

In the subsequent analysis, each river type was further subdivided into multiple subcategories using a clustering approach to capture the finer variations within each type. Specifically, meandering rivers, braided rivers, anastomosing rivers, and anabranching rivers were each divided into three subcategories, while straight rivers were divided into five subcategories, as shown in Fig. 8.

### Spatial distribution of river types

**Fig. 9:**
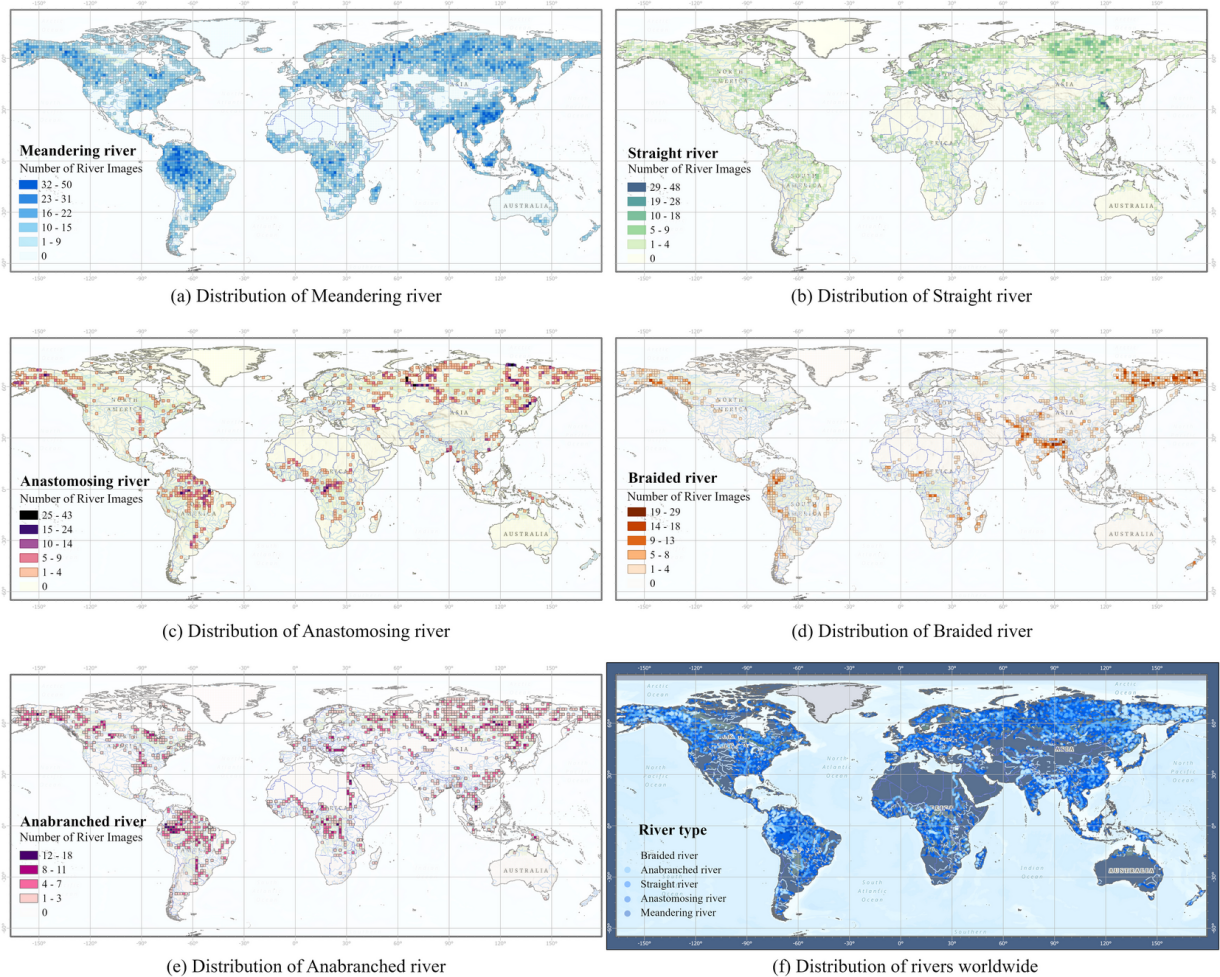
Spatial distribution density maps of five major river types. (**a**) Distribution of meandering river; (**b**) Distribution of straight river; (**c**) Distribution of anastomosing river; (**d**) Distribution of braided river; (**e**) Distribution of anabranched river; (**f**) Distribution of rivers worldwide.

Analyzing the global spatial distribution characteristics of different river types involved extracting geographic coordinates from each river image patch and mapping these onto a global geographic coordinate system. A grid-based analysis method was employed, dividing the globe into regular 1.5° × 1.5° latitude and longitude grids. Subsequently, the number of image patches for each river type was counted within each grid cell. Using this data, spatial distribution density maps were created, detailing the global spatial distribution characteristics for each river type, as illustrated in Fig. 9.

Meandering rivers have a wide global distribution, spanning almost all continents. Straight rivers are primarily distributed in broad plains and regions with intensive human activity. Anastomosing rivers have a relatively limited global distribution but exhibit high-density clusters in specific regions, such as northern Russia, the Bay of Bengal, and the lower Amazon Basin. Braided rivers are predominantly located in regions with significant topographic variation and steep gradients, such as the southern slopes of the Himalayas and parts of the Andes. Anabranching rivers are more dispersed globally but show localized high-density distributions in high-latitude snowmelt recharge zones (e.g., Siberia and northern Canada) and low-latitude, high-sediment basins (e.g., the Amazon Basin and the Mekong River Basin).

**Fig. 10:**
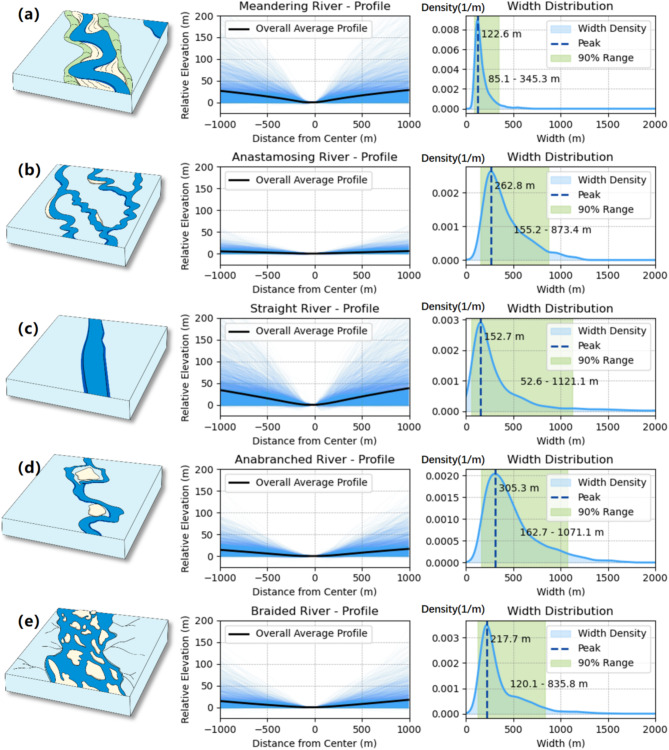
Mean bank elevation and width distribution of five major types of rivers. (**a**) Elevation and width distribution on both sides of the meandering river; (**b**) Elevation and width distribution on both sides of the anastamosing river; (**c**) Elevation and width distribution on both sides of the straight river; (**d**) Elevation and width distribution on both sides of the anabranched river; (**e**) Elevation and width distribution on both sides of the braided river.

Figure 10 illustrates the average bank elevation and width distribution characteristics of the five major river types. Generally, the bank elevations of meandering rivers and straight rivers are significantly higher than those of other river types. For meandering rivers, the higher bank elevations may reflect the dominant lateral erosion processes that carve into the surrounding terrain, creating steep, well-defined banks. In contrast, braided rivers and anabranching rivers typically exhibit lower and more variable bank elevations, indicative of their dynamic sedimentary environments, where frequent deposition and erosion processes reshape the riverbanks.

In terms of width distribution, meandering rivers have the narrowest widths and the most concentrated distributions, reflecting their single-channel morphology and the stable flow conditions of low-gradient river valleys. Straight rivers have slightly wider widths and greater variability. Anastomosing rivers, on the other hand, exhibit a broader width distribution, reflecting the complexity of their multi-channel intersecting systems.

## Discussion

The spatial distribution and morphological characteristics of global river systems are shaped by complex interactions between environmental, geomorphological, and hydrological factors. The findings of this study contribute to a deeper understanding of fluvial geomorphology at a global scale, providing insights into its influencing factors.

Meandering rivers predominate, accounting for 69.78% of the classified dataset and 32,676 image blocks. The predominance of meandering rivers in global river systems is consistent with the existing literature on the geomorphic characteristics of rivers^42^. Leopold and Wolman highlighted the geomorphic conditions that favor the formation of meandering rivers^2^. The high banks of meandering rivers typically result from dominant lateral erosion processes, indicating that the river continuously adjusts its morphology through erosion and deposition, shaping distinct riverbank features.

Anastomosing rivers account for 6.90% of the dataset and 3,232 image blocks, exhibiting high-density clusters in specific regions such as northern Russia, the Bay of Bengal, and the lower Amazon Basin. They are strongly influenced by sediment transport mechanisms and cohesive clayey sediments. These rivers are often observed in areas characterized by gentle terrain, stable hydrological conditions, and depositional environments. Studies on the Apure River in Venezuela have demonstrated that cohesive sediments play a critical role in maintaining the multi-channel morphology of anastomosing systems by providing stability and reducing channel avulsion^43^. These findings highlight the importance of sediment characteristics in sustaining the unique morphology of such rivers.

Braided rivers, which represent 6.06% of the dataset and 2,840 image blocks, are primarily distributed in regions with significant topographic variation and steep gradients, such as the southern slopes of the Himalayas and parts of the Andes. These areas experience intense sediment transport and erosion processes, which actively shape braided channels. For instance, the Brahmaputra River exhibits a complex braided pattern in its midstream reaches due to high sediment loads and dynamic hydrological conditions^14,16^. The spatial distribution of braided rivers reflescts the geomorphological connectivity between sediment source regions (e.g., the Himalayas) and downstream deposition zones, emphasizing the role of sediment supply and steep slopes in their formation.

Anabranching rivers, which account for 8.89% of the dataset and 4,164 image blocks, exhibit a more dispersed global distribution but display localized high-density clusters in specific environmental contexts. High-latitude snowmelt recharge zones, such as Siberia and northern Canada, and low-latitude sediment-rich basins, including the Amazon and Mekong River basins, provide favorable conditions for their development^44^. Factors such as abundant water supply, rich sediment availability, gentle topography, and periodic fluctuations in water levels are critical to the formation and stability of anabranching rivers^36^.

Straight rivers, which account for 8.35% of the dataset and 3,910 image blocks, are primarily distributed in broad plains and regions with intensive human activity. Their slightly wider bank widths and greater variability compared to meandering rivers may be attributed to the inclusion of both natural and artificial channels. These rivers tend to form in environments with relatively high flow energy and low sediment cohesion, which limit the development of complex channel patterns. Their spatial distribution highlights the impact of anthropogenic activities, such as channel straightening for flood management and navigation, on river morphology.

## Conclusion

This study systematically classified global river morphology using a deep learning-based framework and large-scale image-elevation data fusion analysis. By integrating a ResNet-50 deep neural network with a feature fusion module, an innovative classification model was developed, which effectively categorized global rivers into five main types—meandering, straight, anastomosing, braided, and anabranched—and further subdivided them into 17 subtypes. This hierarchical classification framework not only delineated distinct primary types but also revealed fine-grained variations and internal hierarchical structures within each category.

The spatial distribution of global river types exhibits clear geographical patterns. Meandering rivers, the most common type, are widely distributed across all continents. Anastomosing rivers are concentrated in specific regions, such as estuaries and sedimentary environments with gentle terrain. Braided rivers are primarily located in areas with high topographic relief and steep riverbed slopes, such as the southern slopes of the Himalayas. Anabranched rivers show localized high-density distributions in high-latitude snowmelt recharge zones and sediment-rich low-latitude watersheds, such as the Amazon and Mekong basins. These findings provide a detailed understanding of the spatial and geomorphic diversity of global river systems.

This study presents a novel methodological framework for classifying global river morphology, combining supervised classification and unsupervised clustering techniques to capture both macro-scale patterns and fine-grained variations in river morphology. The findings enhance our understanding of fluvial geomorphology, offering new insights into the spatial distribution and structural diversity of river systems. While this research provides a robust tool for analyzing global river morphology, certain limitations remain. For example, the resolution of the datasets may restrict the identification of localized or minute morphological features. Future studies could address these limitations by incorporating higher-resolution datasets and field measurements to further refine the classification framework.

## Data Availability

The Global Inland Water Dynamics Characterization dataset and the global digital elevation model ASTER GDEM V3 used in this study are publicly accessible at https://www.glad.umd.edu/dataset/global-surface-water-dynamics and https://lpdaac.usgs.gov/products/astgtmv003/, respectively. Data generated or processed during this study can be obtained from the corresponding author upon reasonable request.
